# Identifying the Chinese Herbal Medicine Network and Core Formula for Allergic Rhinitis on a Real-World Database

**DOI:** 10.1155/2020/5979708

**Published:** 2020-11-05

**Authors:** Yen-Chu Lu, Ching-Wei Yang, Yi-Hsuan Lin, Ju-Yu Hsueh, Jiun-Liang Chen, Sien-Hung Yang, Yu-Chun Chen, Hsing-Yu Chen

**Affiliations:** ^1^Division of Chinese Internal Medicine, Center for Traditional Chinese Medicine, Chang Gung Memorial Hospital, Taoyuan, Taiwan; ^2^School of Traditional Chinese Medicine, College of Medicine, Chang Gung University, Taoyuan, Taiwan; ^3^Graduate Institute of Clinical Medical Sciences, College of Medicine, Chang Gung University, Taoyuan, Taiwan; ^4^Department of Public Health, China Medical University, Taichung, Taiwan; ^5^School of Medicine, National Yang-Ming University, Taipei, Taiwan

## Abstract

**Materials and Methods:**

Patients with a primary diagnosis of AR (ICD-9-CM code: 477.9) in 2010 were included, and the National Health Insurance Research Database in Taiwan was used as the data source. Association rule mining and social network analysis were used to establish and explore the CHM network. Possible molecular pathways of the CHM network were summarized and compared with commonly used western medicine (WM) by conducting overrepresentation analysis in the Reactome pathway database. The potential proteins acted by CHMs were obtained from the CHM ingredient-protein databases, including STITCH, TCMSP, TCMID, and TCM@Taiwan.

**Results:**

There were 89,148 AR subjects found in 2010, and a total of 33,507 patients ever used CHM. On an average, 5.6 types of CHMs were utilized per prescription. Xin-Yi-Qing-Fei-Tang was used most frequently (25.5% of 222,279 prescriptions), while Xiao-Qing-Long-Tang with Xin-Yi-San was the most commonly prescribed CHM-CHM combination. Up to six distinctive clusters could be found among the CHM network, and core CHMs could be found for AR, such as Xiao-Qing-Long-Tang and Xin-Yi-Qing-Fei-Tang. A total of 140 molecular pathways were covered by the CHM network (2,432 ingredients from 31 kinds of CHMs), while 39 WMs covered 55 pathways. Among pathways responding to the immune system, WM mainly acted on cytokine signaling-related pathways, while CHM mostly acted on neutrophil/macrophage-related innate pathways and dendritic cell-related adaptive immunity pathways.

**Conclusion:**

Our study demonstrated and analyzed the CHM network for AR. Core CHM for AR and possible molecular pathways were presented as well, and this information is crucial for researchers to select candidates for CHM-related studies.

## 1. Introduction

Allergic rhinitis (AR) is a significant global health problem affecting 10%–25% of the population and over 500 million patients worldwide [1, 2]. In Taiwan, the prevalence of AR is around 35% in schoolchildren and shows an increasing trend over the past three decades like other countries [2–4]. AR patients commonly present with paroxysms or perennial of rhinorrhea, nose obstruction, nasal itching, and sneezing in varying degrees and often suffer from AR-related complications, including postnasal drip, night cough, insomnia, irritability, and fatigue [2, 5]. On the other hand, comorbidities such as asthma and atopic dermatitis are also associated with AR as parts of “allergic march,” and therefore, the coexistence of AR, atopic dermatitis, and asthma are not uncommon [6, 7]. Although generally AR is not a fatal disease, it significantly affects the quality of life, social life, learning performance, and work productivity of patients [8–11]. Advances in AR management are still needed [2, 12].

In addition to western medicine (WM), the use of Chinese herbal medicine (CHM) is not uncommon, and the respiratory system and allergic diseases are both the primary reasons to use CHM [13, 14]. For the concept of “cure the root of disease,” the concern about side effects of treatments and desire for controlling AR better, TCM managements have been used for allergic diseases increasing yearly [14–16]. Several clinical studies showed that CHM might be beneficial [17]. Nevertheless, CHM prescriptions are rather complicated to find out the most crucial CHM in clinical studies; for example, more than five kinds of CHM used in a clinical trial were not uncommon, and the medicines immensely varied from trials to trials [17, 18]. This fact causes difficulties in exploring the mechanisms of CHM, applying into clinical practice, undergoing large-scale clinical trials [18, 19].

For this reason, it is vital to analyze the prescription patterns to find out the core part of CHMs for AR. Kung et al. reported the most commonly used single CHM and the CHM-CHM combinations by using Taiwan's clinical data in 2002 [20]; however, we found that the relations between CHMs are usually like a network, rather than simple CHM-CHM connections, and the graphic demonstration of CHM network would present a clear view of CHMs for diseases [21]. Additionally, the role of each CHMs could be found by studying the relationships between CHMs in the network. For example, higher prevalence and connections of a CHM indicate the vital importance of a specific disease, which is similar to pick essential protein in the complicated protein-protein interaction network [21, 22]. Furthermore, applying the modern concepts of network pharmacology to explore the relations among CHM, multiple ingredients of CHM, target proteins, and even potential molecular pathways would help integrate the latest pharmacology mechanisms (such as anti-inflammation, antiallergic, and antioxidation effects for allergic skin disease) and the conventional TCM viewpoints for CHM (for example, qi-deficiency and wind and dampness syndrome for allergic diseases) into a single disease CHM network. Consequently, an integrative and in-depth overview of CHMs used for a specific disease could be presented and, more importantly, understandable [23–25].

This study aims to demonstrate the CHM network, explore the core CHMs, and explore the possible pharmacology mechanisms' CHMs for AR by connecting the CHM network to biomedicine databases in the network pharmacology approach. Additionally, we also compared the proposed molecular pathways covered by the CHM network with all kinds of WMs for AR to assume the different roles of WM and CHM in treating AR. The results of this study would enhance the understating of CHM for AR for both WM and TCM researchers.

## 2. Materials and Methods

### 2.1. Data Source

The National Health Insurance Research Database (NHIRD), with high coverage (>99%) of medical records from all inhabitants enrolled in the National Health Insurance program in Taiwan since 1995, was used as the data source for this study. This database included comprehensive information about each patient's medical utilization and demographic features, such as in-patient and out-patient management, drop-off prescriptions, patients' gender, birth date, comorbidities, living space, medical expenditure, examinations, biochemical tests, reasons to visit/admission, and insured level. The NHIRD contains nearly all interventions made by qualified specialists, including WM, TCM doctors, and dentists, and therefore becomes an unequaled and suitable data source for observational studies [21, 26, 27]. For privacy protection, all patients' identification numbers are well encrypted, and therefore, it is not possible to recognize everyone's identity. The NHIRD is especially crucial for TCM-related studies since Taiwan is one of the few countries that fully reimburses all TCM treatment modalities, including CHM, acupuncture, and manual therapy. Therefore, the NHIRD becomes a comprehensive database with sufficient information for clinical studies [26].

### 2.2. Study Design

**Figure 1:**
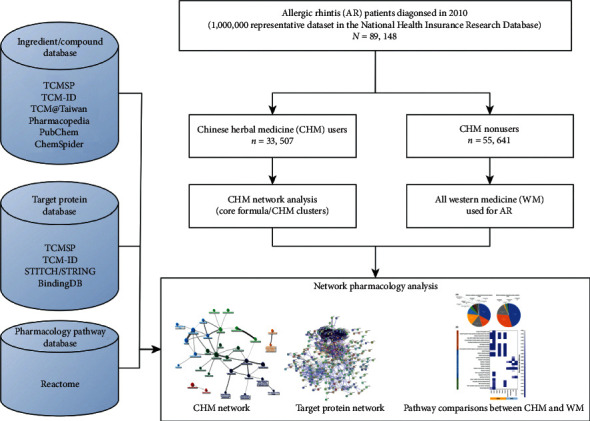
Flow diagram of this study.

Figure 1 shows the flow diagram of this study. A one-million subjects' representative database was sampled from the entire Taiwan population in 2010 to retrieve CHM prescription data, and the International Classification of Diseases, Ninth Revision, Clinical Modification (ICD-9-CM) 477.9, was used to identify AR patients. As the latest guideline, AR is primarily diagnosed by three typical symptoms as guideline suggestions, including sneezing, nasal obstruction, and running nose [2, 28]. Although using ICD-9-CM code to detect the reasons for medical utilization and comorbidities is reliable and well acceptable, only patients with a primary diagnosis of AR were included in the analysis to make enrollment as precise as possible [21, 27, 29]. Furthermore, CHM prescriptions were collected from all eligible subjects, and whoever used CHM once was recognized as a CHM user, while AR patients who never used CHM were classified as CHM nonusers. Patients and their visits with the management of acupuncture, moxibustions, and manual therapies were excluded. The institutional review board of the Chang Gung Memorial Foundation approves the study protocol (No. 202000248B1).

### 2.3. CHM Prescription Database

Two types of CHMs were contained in the CHM prescription database: herbal formula (HF) and single herb (SH). Every SH was a kind of concentrated powder made from either part of natural plants, animals, minerals, and insects according to the preparation methods suggestions in TCM pharmacopeia. On the other hand, HF was a mixture of more than one kind of SH with fixed proportion according to the CHM classics, and the mixes were performed in the pharmaceutical factories before selling. In this study, pharmaceutical names were used to present SHs by searching the Plant List website (http://www.theplantlist.org/), and Chinese names in pinyin were used to present HFs, followed by lists of SHs. Additionally, all pharmaceutical factories must conform to the regulation of good manufacturing practice (GMP), which has been zero tolerance to heavy metal, pesticide, or even known toxins, such as aristolochic acid.

Furthermore, the composition of CHM preparations was acquired from the webpage provided by the Ministry of Health and Welfare in Taiwan, which has been the authority of the marketing and quality control of all CHMs (https://dep.mohw.gov.tw/DOCMAP/lp-874-108.html) (supplementary materials S1). Also, the ingredients of every CHMs were retrieved from TCM pharmacopeia and three databases, including the Traditional Chinese Medicine Systems Pharmacology (TCMSP), TCM Database@Taiwan, and the Traditional Chinese Medicine Integrated Database (TCMID) [30–32]. To select the most eligible ingredients, we adopted the suggestions about ingredient screening from TCMSP. The drug-likeness value should be higher than 0.18 and bioavailability higher than 0.30, and the inorganic compounds and ingredients least like drugs were excluded (supplementary materials S2).

### 2.4. Statistical Analysis

Two parts of statistical analysis were performed. First, descriptive statistics were used for presenting baseline features of TCM users, such as age, gender, comorbidities, insured level, living locations, and coexisting WM and comorbidities, and these features were compared with CHM nonusers. Second, association rule mining (ARM) and social network analysis (SNA) were both used to construct the CHM network for AR. The data processing protocol was presented in our previous works about the CHM network for allergic skin diseases [21]. In short, ARM was used to evaluating the importance of all possible CHM-CHM combinations at first by using three parameters: support, confidence, and lift. Single CHM with higher support meant a higher prevalence of single CHM among all prescriptions. In comparison, more top support for CHM-CHM combinations presented a higher prevalence of CHM-CHM combinations. Additionally, confidence and lift were both representatives of the strength of CHM-CHM combinations, and higher values showed more robust connections between two CHMs [21]. Thresholds for these three parameters were preset, and use our previous works for allergic skin diseases [21].

The top 100 CHM-CHM combinations were further used to construct the CHM network and analyzed by SNA for every CHM role in the CHM network. By analyzing the relations of CHMs in the network, CHMs could be grouped into different clusters, in which CHM with closer relationships would be grouped [33, 34]. Moreover, the CHM prevalence and degree centrality were used to present each CHM's role in the network. Degree centrality, as the total number of connections to a node, was commonly used to recognize the importance of every node on the entire network. Nodes with higher degree centrality represented higher importance among all CHMs, and degree centrality was used to find out study candidates in biomolecular networks [22]. Likewise, since TCM doctors usually composed CHM prescriptions by picking some sorts of CHM as the core, then adding on other CHMs, the CHM with higher prevalence and higher degree centrality may indicate the more crucial role in the CHM network [21].

Furthermore, CHM indications and possible molecular mechanisms were incorporated into the CHM network, and the mechanisms of WM were obtained to broaden the understanding of the CHM network from both TCM and WM viewpoints. The WM for AR were collected from the latest guideline and listed in the supplementary materials S3. CHM indications were obtained and summarized from CHM pharmacopeia [21]. On the other hand, to summarize the molecular mechanisms of CHMs, we dissected every CHM to a pure compound level. We then obtained the possible target proteins by using freely accessible databases, including STITCH and BindingDB [24, 35, 36]. Then, the target proteins of each cluster were used to propose the possible molecular mechanisms by using overrepresentation tests in Reactome, a freely accessible web resource to estimate, interpret, and visualize the molecular mechanisms of a given group of genes or proteins [37]. As external validation, each CHM was searched manually in PubMed for possible molecular mechanisms for AR.

The freeware KNIME (version 3.4) was used to deal with the databases, and NodeXL was used to build up the networks and perform SNA. The commercial statistical software STATA (Release 16. College Station, TX) was used to carry out the descriptive statistical analysis. Statistics with a *p* value ≤0.05 represented significant results.

## 3. Results

### 3.1. Features of CHM Users

A total of 33,507 patients with AR were identified as CHM users, while 55,641 CHM nonusers were found (Table 1). There were more female patients among CHM users (56.4% versus 47.4% among CHM nonusers, *p* value <0.001). Although the causality was unable to be ensured due to the cross-sectional design of this study, we found that patients with allergic comorbidities such as atopic dermatitis (CHM users versus nonusers: 17.8% versus 16.7%, *p* value <0.001) and chronic sinusitis (CHM users versus nonuser: 3.9% versus 3.1%, *p* value <0.001) tended to use CHM. Most people used more than two kinds of WM (54.4% of all subjects), and this trend was more prominent among CHM nonusers (50.1% for CHM users versus 57.0% for nonusers, *p* value <0.001).

### 3.2. Prevalence of CHM Commonly Used for AR

**Figure 2:**
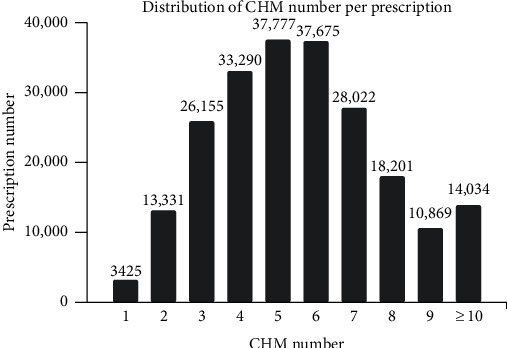
The distribution of Chinese herbal medicine (CHM) counts per prescription.

There were 222,279 CHM prescriptions made for AR in 2010. TCM doctors usually prescribed multiple CHMs for allergic rhinitis concomitantly, and there were 5.6 CHMs used in one prescription on an average. Most prescriptions were composed of 5 CHMs (17% of all prescriptions), and 6.3% of prescriptions contained at least ten kinds of CHMs (Figure 2). Among all HFs, Xin-Yi-Qing-Fei-Tang was prescribed most frequently (25.5% of all prescriptions) (Table 2), followed by Xiao-Qing-Long-Tang (22.9%), Xin-Yi-San (20.2%), Cang-Er-San (18.4%), and Ge-Gen-Tang (17.7%) (the compositions of HFs are listed in the supplementary materials S1 and S2). On the other hand, *Platycodon grandiflorum* (Jacq.) A. DC. (19.1%) was the most prescribed SH of all prescriptions, followed by *Glycyrrhiza uralensis* Fisch. (16.5%), *Angelica dahurica* Benth. et Hood. F. (16.2%), *Scutellaria baicalensis* Georgi (15.8%), and *Fritillaria thunbergii* Miq. (15.5%) (Table 3). The prevalence of commonly used HFs was nearly higher than SHs, and the average dosage of HFs was around 4-5 gm/day, which was 3-4 times higher than SHs (1–1.5 gm/day) (Tables 2 and 3).

### 3.3. Analysis of the CHM Network for AR

Furthermore, CHM-CHM combinations were explored to build up the CHM network, and the top 10 commonly used CHM-CHM combinations are listed in Table 4 as an example. Xiao-Qing-Long-Tang combined with Xin-Yi-San was used most frequently (support: 1.955, confidence: 23.319, lift: 2.488), followed by Xin-Yi-Qing-Fei-Tang combined with *Houttuynia cordata* Thunb. (support: 1.511, confidence: 25.620, lift: 2.274), *Saposhnikovia divaricata* (Turcz.) Schisch. combined with *Schizonepeta tenuifolia* (Benth.) Briq. (support:1.364, confidence: 35.586, lift: 8.628), *Glycyrrhiza uralensis* Fisch. combined with *Platycodon grandiflorum* (Jacq.) A. DC. (support: 1.337, confidence: 17.782, lift: 2.202), and *Platycodon grandiflorum* (Jacq.) A. DC. combined with *Fritillaria thunbergii* Miq. (support:1.298, confidence: 21.082, lift: 2.803).

**Figure 3:**
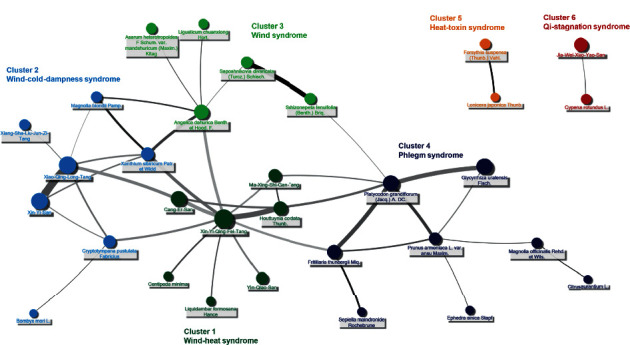
Chinese herbal medicine (CHM) network for allergic rhinitis (AR).

Figure 3 demonstrates the CHM network by using the top 100 commonly used CHM-CHM combinations. This network presents a comprehensive overview of CHM for AR, in which larger circles mean a higher prevalence of single CHM, and thicker and darker edges between CHMs represent more prevalent and more reliable connections, respectively. By applying SNA, these CHMs were divided into six clusters according to the relations between CHMs. By incorporating indications to each CHM according to TCM theory, each cluster had its tendency to treat specific TCM syndrome, which is represented by different colors in Figure 3. For example, cluster 1 contained CHMs referring to AR patients with the wind-heat syndrome, with Xin-Yi-Qing-Fei-Tang as its core part; cluster 2 tended to treat wind-cold-dampness syndrome among AR patients with Xiao-Qing-Long-Tang as its core part. Clusters 3–6 were composed of SHs mainly and less frequently used than HFs. For instance, cluster 3 was indicated to wind syndrome by combining SHs with *Angelica dahurica* Benth. et Hood. F. Moreover, some CHMs were isolated from other clusters but formed strong connections between each other. For example, *Forsythia suspensa* (Thunb.) Vahl. and *Lonicera japonica* Thunb. composed cluster 5, and Cyperus rotundus L. and Jia-Wei-Xiao-Yao-San composed cluster 6.

### 3.4. Proposed Molecular Pathways of the CHM Network and WM

To explore the mixed pharmacological effects of the CHM network with consideration of CHM-CHM combinations and examine the differences in molecular mechanisms between CHM and WM, we investigated possible actions of CHMs and WMs by using the abovementioned methods. A total of 39 kinds of WMs and 31 kinds of CHMs (with 2,432 ingredients) were used to find their target proteins. All WMs are listed in the supplemental material S3, and the compositions of HF preparation and ingredients are provided in the supplementary materials S1 and S2, respectively. Supplementary material S4 shows all possible binding proteins of every CHM and WM.  Table 5 reveals the overlapped binding proteins associated with the immune system between WMs and CHMs. CHMs shared only a relatively small proportion of binding proteins with WMs, and these proteins seemed related to inflammation, such as FOS, TNFA, and MMP9. Among 6 CHM clusters, only clusters 1 and 2 covered binding proteins across all four categories of WMs.

**Figure 4:**
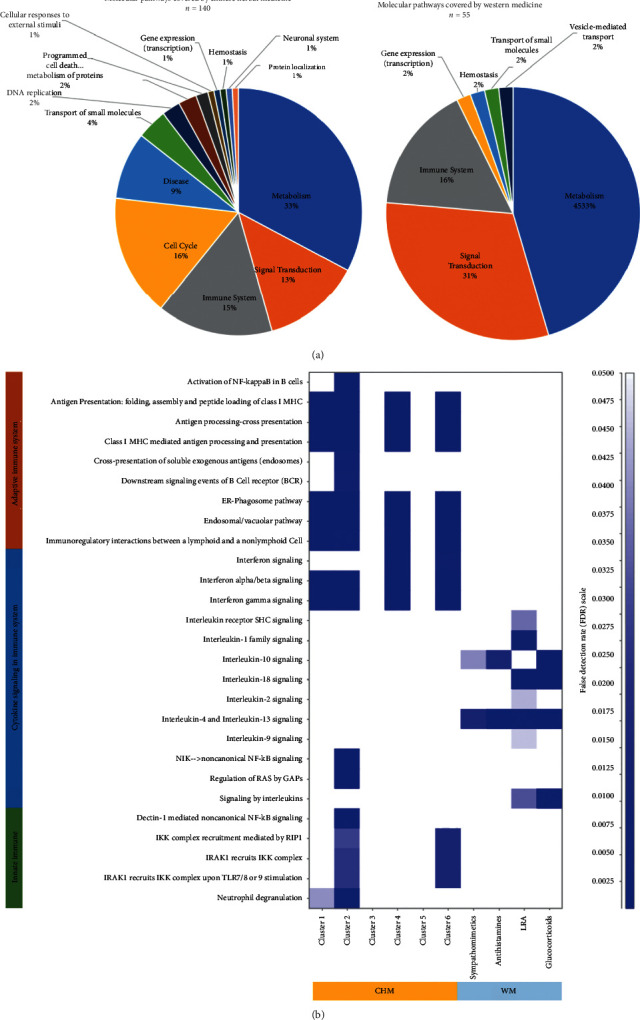
The molecular pathways covered by Chinese herbal medicine (CHM) and western medicine (WM). (a) The distribution of covered pathway categories among CHM (left) and WM (right). (b) Different coverage patterns of immune system-related pathways among different clusters of CHM and WM.

Also, the potential molecular pathways of CHM clusters and WM were proposed by considering binding proteins within every cluster and seemed quite different (Figure 4(a), the details listed in the supplementary material S5). Overall, we found 140 molecular pathways covered by 31 CHMs in the network, while 55 molecular pathways were covered by 39 WMs, and the actions of molecular pathways were more diverse among CHMs. For the immune system, CHMs covered 19 molecular pathways, while WMs covered eight. Interestingly, the involved pathways seemed quite different between CHMs and WMs, and mutually complementary effects could be observed (Figure 4(b)). WMs mainly acted on cytokine signaling-related pathways, while CHMs mostly acted on immune cells with innate and adaptive immunity-related pathways. Even in the cytokine signaling category, the complementary effects were found that CHMs acted on interferon-related pathways, and WMs mainly acted on interleukin-related pathways.

As external validation, we manually searched works of literature of CHMs in the network (Table 6, last assessed date at PubMed: 2020/5/31). Few commonly used HFs or SHs have shown possible pharmacological effects on AR. As for the immune system, only Xiao-Qing-Long-Tang and Xin-Yi-San have changed the cytokines level among AR patients, and anti-inflammation effects were commonly provided by other CHMs (Table 6).

## 4. Discussion

In this study, we illustrated the CHM network to present core CHMs for AR and proposed the potential pharmacological mechanisms by utilizing freely accessible biomedicine databases to analyze the real-world clinical database. For treating AR, multiple CHMs used in one prescription are as common as our earlier reports about CHM network allergic skin diseases [21]. This phenomenon well reflects the complexity in AR pathophysiology on WM's viewpoint and the principle of composing CHM prescription for AR. TCM has a unique philosophy for composing prescriptions, which primarily depends on the diagnosis of TCM syndrome, a summary of the patient's clinical symptoms, and cause of disease according to TCM diagnosis theory, rather than pathophysiology on WM's viewpoints on AR. After syndrome diagnosis, “Sovereign, minister, assistant, and courier” is the mainstay idea of prescription composing, which means TCM doctors would choose some CHMs as the core of prescription and then add on other CHMs to augment or antagonize the effects of core CHMs for patient's syndrome. Therefore, the use of the CHM network may provide a feasible model for understanding TCM prescriptions [21, 60].

Through analyzing the CHM network, several implications could be explored: (1) the primary “TCM syndromes” that CHMs used to treat AR, (2) the different roles of CHM and WM on managing AR, (3) core CHM formula, and (4) unique CHM-CHM combinations. TCM syndrome could be a concise summary of disease, and recognition of TCM syndrome is vital to understand the principle of CHM treatments. By incorporating indications to CHMs, the preferable TCM syndrome for AR could be proposed. Wind-cold-dampness syndrome (cluster 2) and wind-heat syndrome (cluster 1) seemed the primary syndromes for CHM, and this trend is also observed in clinical trials [60, 61]. The presentation of the wind-cold-dampness syndrome includes clear nasal discharge, stuffy nose, and itchy nose, and these symptoms are usually precipitated by cold exposure [62]. On the contrary, patients with wind-heat syndrome were often diagnosed by yellowish nasal discharge and nasal obstruction, which is like rhinosinusitis, and this may correspond to the high coexistence of AR and sinusitis.

In addition to TCM syndrome, CHMs seemed to have special pharmacological effects as mutually complementary therapies to WM for AR. Overall, CHMs covered more molecular pathways when compared to commonly used WMs. The far larger number of identifiable pure compounds among CHMs may be the primary reason (2,398 pure compounds from all CHMs vs. 39 kinds of WM). Although most TCM doctors prescribe CHMs for AR based on TCM theory, rather than pharmacological mechanisms at the molecular level, the molecular pathways proposed by each CHM cluster uncover the reasons why these CHMs were chosen and rationales of CHM combinations. When focusing on immune system-related molecular pathways, the CHM clusters covered mainly pathways involved in the innate and adaptive immune systems; in contrast, WM mostly acted on cytokine signaling pathways, primarily interleukin-related actions (Figure 4(b)). According to Reactome's classifications, the adaptive immune system includes pathways related to antigen-presenting cells, the innate immune system contains neutrophil and macrophage-related pathways, and cytokine signaling pathways are composed of cytokines and their intracellular downstream pathways [37]. Conventionally, AR has been thought of as an IgE-mediated response; nevertheless, the importance of neutrophil and macrophage for AR has been rising recently. Among AR patients, higher neutrophil correlates to more severe AR symptoms, and neutrophil has been a significant factor in copious amounts of secretion and epithelial disruption [63–65]. However, glucocorticoids may affect the survival of neutrophils and then not effective among all AR patients [66]. This population of AR patients may be good candidates for CHM treatment for high coverage over the innate immune system. Moreover, recent reports found that macrophage activation and migration played an essential role in precipitating allergic symptoms, and the macrophage migration inhibitory factor (MIF) was found crucial in this process [67, 68]. We also found some CHMs may act on MIF and FOS, which are both essential for inflammation response [67, 69] (Table 5).

Although CHMs seemed to have some effects other than WMs, the exploration of core CHM for AR is still needed since CHM prescriptions are often too complicated to understand. Using SNA for the CHM network, we proposed core CHM for each cluster, with high degree centrality and prevalence. As for wind-heat syndrome (cluster 1), Xin-Yi-Qing-Fei-Tang was the core formula and strongly connected to *Houttuynia cordata* Thunb. They shared similar mechanisms such as anti-inflammation and antibacterial effects. Xin-Yi-Qing-Fei-Tang can reduce the nasal colonization of *Streptococcus pneumonia,* which causes sinusitis and increases tumor necrosis factor-alpha (TNF-*α*), interleukin-1 beta (IL-1*β*), interleukin-6 (IL-6), and monocyte chemotactic protein-1 (MCP-1) levels and the migration of macrophages [38]. *Houttuynia cordata* Thunb. shows antibacterial effects against methicillin-resistant *Staphylococcus aureus* (MRSA) and antibiofilm activity against MRSA and induced interleukin-8 (IL-8) [70]. Microbial infection is one of the leading causes of sinusitis or relapsed AR, but the long-term use of antibiotics may raise the concern about the generation of antibiotic-resistant strain. Enhancing immunity rather than directly eliminating microbial by using CHM becomes an available treatment option for AR.

On the other hand, for the wind-cold-dampness syndrome (cluster 2), Xiao-Qing-Long-Tang with the highest prevalence and degree centrality seemed to be the core formula. The interconnections between CHMs reflected the principle of composing CHM prescription, the so-called “sovereign, minister, assistant, and courier.” In this case, as the core formula, Xiao-Qing-Long-Tang dispels the wind-cold and dampness (“sovereign,” the main actor of a formula), and the effect can be strengthened by adding Xin-Yi-San as the “minister” part of the formula for patients with more cold-caused symptoms, such as nasal obstruction or rhinorrhea. Besides, the other CHMs in cluster 2 may serve as “assistant” and “courier,” which means optional CHMs to specific conditions, such as Xiang-Sha-Liu-Jun-Zi-Tang for patients with more dampness and Cang-Er-San for patients with possible heat transformation, which is similar to AR complicated with sinusitis. The principle of combing these CHMs is quite reasonable from the pharmacological viewpoint. Xiao-Qing-Long-Tang helps the body regulate T cell functions, and the immunomodulation on the adaptive immune system may be enhanced when combining Xiao-Qing-Long-Tang with Xin-Yi-San and Xiang-Sha-Liu-Jun-Zi-Tang [45, 71, 72].

Additionally, “drug pair” is a kind of unique CHM-CHM combination noted in the CHM network (clusters 5 and 6 in Figure 3). For example, *Forsythia suspensa* (Thunb.) Vahl. and *Lonicera japonica* Thunb. were positively associated with each other but had no relations to other CHMs in the network. Both CHMs can clear heat and detoxify, and they were essential CHMs for treating allergic skin disease or acne due to their profound anti-inflammatory effects [19, 21, 73].

There were some similarities and differences between this study and previous studies about CHM prescriptions for AR. Kung et al. and Lin et al. reported the common single CHM and CHM-CHM combinations for AR in Taiwan with different time frames in our study [14, 20]. These results were quite similar to our findings, which may indicate the CHM prescriptions for AR were stable in recent decades, and the results in our study were reliable. On the other hand, some studies focused on single HF, such as Ma-Huang-Fu-Zi-Xi-Xin-Tang [74] and Xiang Ju tablet [75], or single SH, such as Aconiti lateralis Radix Praeparata [76], Flos magnolia, and *Centipeda minima* [77]. Overall, even though the molecular pathway databases were KEGG (Kyoto Encyclopedia of Genes and Genomes) in these studies instead of Reactome in our work, anti-inflammation effects have been widely discovered among CHMs used for AR, and IL-6, IL-8, and TNF-*α* were the common target proteins. However, from the CHM network perspective, the influence on the immune system would be more extensive when combing certain CHMs. By referencing to Reactome database, more detailed molecular pathways could be found in both adaptive and innate immune systems.

Using the real-world data and CHM network analysis, we can graphically demonstrate the CHM prescriptions for AR, and we first connect the freely accessible biomedicine databases and CHM network to the proposed potential effects of CHM freely. Relying on the nature of the NHIRD, the high coverage of Taiwan inhabitants, and well-established electronic medical records, selection bias and recall bias could be minimized, and these results could be regarded as a sort of consensus summarized from real-world clinical data. However, there are still some drawbacks to this study. First, since the CHM prescriptions are obtained from the Taiwan clinical database, the generalizability would be a problem since the differences in environments or patients' constitution may influence the CHM prescribed for AR patients. Nevertheless, we provided a possible way to connect clinical data to biomedical databases, and the effects of CHMs on AR would be more exact if more studies from other places are conducted in the future. Second, there were no data about TCM syndrome or AR symptoms recorded in this database. Although we can propose preferable TCM syndrome by analyzing the CHM network, comprehensive data collection about TCM syndrome is still needed [78]. The result of this study would become a key recommendation on the coding system used for data collection. Third, there were no lab data or symptom severity recorded in the NHIRD, and this work is a cross-sectional study in design. Therefore, it is neither feasible to precisely categorize AR patients as WM guidelines suggested or examine the efficacy of CHM for AR due to the nature of this study. However, we found that some of the core CHMs have been found useful for AR by clinical trials, such as Xiao-Qing-Long-Tang, Xin-Yi-San, and Xiang-Sha-Liu-Jun-Zi-Tang, which show the potential of other core CHM for AR. Well-designed clinical trials and TCM syndrome assessment may be needed to solve this problem. Fourth, the possible molecular pathways proposed in this study were mainly obtained from publicly available biomedical databases, which gathered an enormous amount of information in many ways, including text mining, animal or cell experiments, and inferences from species other than human; therefore, the experiments to validate the mechanisms of CHM are still needed. Last, the dose of CHM was not considered when we performed pathway analysis, and this may cause biases when exploring potential molecular pathways. Although we treated every CHM and its ingredients equally in this study, we only selected drug-liked ingredients and their target proteins for exploring potential molecular pathways. Bench experiments are still needed to study the dose-efficacy relations between CHM ingredients and AR-related molecular pathways. Nevertheless, we could provide the average daily dose of CHMs for AR from the clinical practice perspective. Although the ingredients' concentration of every CHM may be different between different pharmaceutical factories, and it makes the estimation of ingredients' concentration difficult. The daily dose of CHM used for AR could be future references to choose ingredients concentration in bench experiments.

## 5. Conclusion

We graphically demonstrated the CHM network for AR in this study, and by connecting the CHM network to the biomedicine database and comparing the possible molecular pathways of CHM with WM, the complementary roles of CHM and WM are proposed as well. The results would broaden the horizon on using CHM and WM for AR, and it would become an essential reference for further clinical studies and experiments.

## Figures and Tables

**Table 1:** Characteristics of Chinese herbal medicine (CHM) users and nonusers among patients with allergic rhinitis (AR) in Taiwan in 2010 (*N* = 89,148).

	CHM users, *n* = 33,507		CHM nonusers, *n* = 55,641		*p* value
Gender					
Male	14,607	(43.6%)	29,276	(52.6%)	<0.001
Female	18,900	(56.4%)	26,365	(47.4%)	—

Age, years					
0–20	12,572	(37.5%)	22,941	(41.2%)	<0.001
21–40	10,350	(30.9%)	12,869	(23.1%)	—
41–	10,585	(31.6%)	19,831	(35.6%)	—

Urbanization level					<0.001
1 (most urbanization)	19,229	(57.4%)	33,689	(60.5%)	—
2	11,362	(33.9%)	17,205	(30.9%)	—
3 (least urbanization)	2,916	(8.7%)	4,747	(8.5%)	—

Insured level (in NTD per month)					<0.001
1–19,999	21,110	(63.0%)	37,326	(67.1%)	—
20,000–39,999	8,485	(25.3%)	12,242	(22.0%)	—
40,000–	3,912	(11.7%)	6,073	(10.9%)	—

Combined allergic disease					
Atopic dermatitis	5,957	(17.8%)	9,275	(16.7%)	<0.001
Asthma	4,178	(12.5%)	9,836	(17.7%)	<0.001
Chronic sinusitis	1,311	(3.9%)	1,720	(3.1%)	<0.001

Conventional treatment					
1^st^ antihistamine	10,614	(31.7%)	18,496	(33.2%)	<0.001
2^nd^ antihistamine	26,280	(78.4%)	49,693	(89.3%)	<0.001
Decongestants	8,384	(25.0%)	13,229	(23.8%)	<0.001
Intranasal antihistamine	157	(0.5%)	422	(0.8%)	<0.001
Intranasal corticosteroid	6,156	(18.4%)	14,587	(26.2%)	<0.001
Leukotriene receptor antagonists	1,095	(3.3%)	2,723	(4.9%)	<0.001

Types of medications					
0	5,097	(15.2%)	2,672	(4.8%)	<0.001
1	11,613	(34.7%)	21,234	(38.2%)	—
2	10,691	(31.9%)	19,975	(35.9%)	—
≥3	6,106	(18.2%)	11,761	(21.1%)	—

NTD, new Taiwan dollar (exchange rate: about 30 NTD–1 USD).

**Table 2:** The top 5 commonly prescribed herbal formulas (HFs) for allergic rhinitis (AR) (prescription number: 222,279).

Rank	HF name	Composition (% w/w)	CHM indications	Prevalence (%)	Dose (gm/day)	Degree centrality
1	Xin-Yi-Qing-Fei-Tang	*Anemarrhena asphodeloides* Bge. (11.9%), *Cimicifuga heracleifolia* Kom. (3.6%), *Eriobotrya japonica* (Thunb.) Lindl. (11.9%), *Gardenia jasminoides* Ellis. (11.9%), *Glycyrrhiza uralensis* Fisch. (6.0%), *Gypsum fibrosum* (11.9%), *Lilium brownii* F. E. Brown var. colchesteri Wils. and *Lilium pumilum* DC. (11.9%), *Magnolia biondii* Pamp. (7.1%), *Ophiopogon japonicus* Ker-Gawl. (11.9%), and *Scutellaria baicalensis* Georgi (11.9%)	Wind-heat invading the upper respiratory tract	25.5	4.88	10
2	Xiao-Qing-Long-Tang	*Asarum heterotropoides* F Schum. var. mandshuricum (Maxim.) Kitag (7.9%), *Cinnamomum cassia* Blume (15.8%), *Ephedra sinica* Stapf (10.5%), *Glycyrrhiza uralensis* Fisch. (15.8%), *Paeonia lactiflora* Pall. (10.5%), *Pinellia ternata* (Thunb.) Breit. (15.8%), *Schisandra chinensis* (Turcz.) Baill. (7.9%), and *Zingiber officinale* Rosc. (15.8%)	Wind-cold with dampness in the respiratory system	22.9	5.35	6
3	Xin-Yi-San	*Angelica dahurica* Benth. et Hood. F. (10.0%), *Asarum heterotropoides* F Schum. var. mandshuricum (Maxim.) Kitag (10.0%), *Camellia sinensis* (L.) O. Ktze. (10.0%), *Cimicifuga heracleifolia* Kom. (10.0%), *Clematis armandii* Franch. (10.0%), *Glycyrrhiza uralensis* Fisch. (10.0%), *Ligusticum chuanxiong* Hort. (10.0%), *Ligusticum sinense* Oliv. (10.0%), *Magnolia biondii* Pamp. (10.0%), and *Saposhnikovia divaricata* (Turcz.) Schisch. (10.0%)	Wind-cold invading the nose	20.2	5.23	3
4	Cang-Er-San	*Allium fistulosum* L. (10.5%), *Angelica dahurica* Benth. et Hood. F. (42.1%), *Camellia sinensis* (L.) O. Ktze. (5.3%), *Magnolia biondii* Pamp. (21.1%), *Mentha haplocalyx* Briq. (10.5%), and *Xanthium sibiricum* Patr. et Widd (10.5%)	Wind-heat invading the nose	18.4	4.41	3
5	Ge-Gen-Tang	*Cinnamomum cassia* Blume (11.1%), *Ephedra sinica* Stapf (16.7%), *Glycyrrhiza uralensis* Fisch. (11.1%), *Paeonia lactiflora* Pall. (11.1%), *Pueraria lobata* (Willd.) Ohwi (22.2%), *Zingiber officinale* Rosc. (16.7%), and *Zizyphus jujuba* Mill. (11.1%)	Wind-cold	17.7	5.68	0

**Table 3:** The top 10 commonly prescribed single herbs (SHs) for allergic rhinitis (AR), and pharmaceutical names were used to present the listed SHs (prescription number: 222,279).

Rank	Pharmaceutical name	CHM indications	Prevalence (%)	Dose (gm/day)	Duration (days/year)	Degree centrality
1	*Platycodon grandiflorum* (Jacq.) A. DC.	Phlegm	19.1	1.4	17.7	6
2	*Glycyrrhiza uralensis* Fisch.	Heat-toxin	16.5	1.1	23.1	0
3	*Angelica dahurica* Benth. et Hood. F.	Wind	16.2	1.4	20.3	6
4	*Scutellaria baicalensis* Georgi	Heat	15.8	1.4	18.7	0
5	*Fritillaria thunbergii* Miq.	Phlegm	15.5	1.3	18.7	4
6	*Houttuynia cordata* Thunb.	Heat-toxin	14.8	1.5	18.2	4
7	*Prunus armeniaca* L. var. ansu Maxim.	Phlegm	12.9	1.4	16.5	5
8	*Xanthium sibiricum* Patr. et Widd	Wind-cold	12.8	1.4	19.3	5
9	*Cryptotympana pustulata* Fabricius	Dampness	11.8	1.4	20.4	4
10	*Saposhnikovia divaricata* (Turcz.) Schisch.	Wind	11.5	1.1	17.8	2

**Table 4:** The top 10 commonly used two combined Chinese herbal medicine (CHM) for allergic rhinitis (AR), while A and B represent two different kinds of CHMs.

Rank	CHM A		CHM B	Confidence	Support	Lift
1	Xiao-Qing-Long-Tang	with	Xin-Yi-San	23.319	1.955	2.488
2	Xin-Yi-Qing-Fei-Tang	with	*Houttuynia cordata* Thunb.	25.620	1.511	2.274
3	*Saposhnikovia divaricata* (Turcz.) Schisch.	with	*Schizonepeta tenuifolia* (Benth.) Briq.	35.586	1.364	8.628
4	*Glycyrrhiza uralensis* Fisch.	with	*Platycodon grandiflorum* (Jacq.) A. DC.	17.782	1.337	2.202
5	*Platycodon grandiflorum* (Jacq.) A. DC.	with	*Fritillaria thunbergii* Miq.	21.082	1.298	2.803
6	Xin-Yi-Qing-Fei-Tang	with	Cang-Er-San	16.836	1.227	1.495
7	Xin-Yi-Qing-Fei-Tang	with	*Xanthium sibiricum* Patr. et Widd	22.138	1.128	1.965
8	Xiao-Qing-Long-Tang	with	*Xanthium sibiricum* Patr. et Widd	15.413	1.123	1.644
9	*Platycodon grandiflorum* (Jacq.) A. DC.	with	*Prunus armeniaca* L. var. ansu Maxim.	24.027	1.114	3.195
10	Xin-Yi-Qing-Fei-Tang	with	*Angelica dahurica* Benth. et Hood. F.	16.055	1.068	1.425

Single herbs (SHs) are presented as their pharmaceutical names, and Chinese name is used to present the herbal formula (HF, the premixture of SHs) (prescription number: 222,279).

**Table 5:** The lists of overlapped binding proteins of Chinese herbal medicine (CHM) and western medicine (WM) for immune system-related proteins. The number of all binding proteins of CHM and WM clusters is presented as well (the detail binding proteins of each group are listed in the supplementary materials S4).

	Sympathomimetics (*n* = 55)	Antihistamines (*n* = 32)	LRA (*n* = 7)	Glucocorticoids (*n* = 29)
Cluster 1, wind-heat syndrome (*n* = 356)	FOS, COLI, TNFA, IL-8, and MIF	COLI, IL-8, and MMP9	IL-13	COLI and TNFA
Cluster 2, wind-cold-dampness syndrome (*n* = 480)	FOS, COLI, TNFA, IL-8, and MIF	COLI, IL-8, and MMP9	LOX5 and IL-13	COLI and TNFA
Cluster 3, wind syndrome (*n* = 152)	FOS, IL-8, and MIF	IL-8 and MMP9	—	—
Cluster 4, phlegm syndrome (*n* = 244)	FOS, TNFA, and IL-8	IL-8 and MMP9	—	TNFA
Cluster 5, heat-toxin syndrome (*n* = 104)	FOS and IL-8	IL-8 and MMP9	—	—
Cluster 6, Qi-stagnation syndrome (*n* = 326)	FOS, IL-8, and MIF	IL-6, IL-8, and MMP9	—	IL-6

*∗*LRA, leukotriene receptor antagonist.

**Table 6:** Potential mechanisms of commonly prescribed Chinese herbal medicine (CHM) for allergic rhinitis (AR) (last assessed date from PubMed: 2020/5/31).

CHM	Possible mechanisms
Herbal formula (HF)	
Xin-Yi-Qing-Fei-Tang	Antibacterial effect by activating macrophage in murine sinusitis model [38]
Xiao-Qing-Long-Tang	Antiallergy effect by reducing IL-4, IL-6, and TNF-*α* levels [39]. Anti-inflammation by suppressing NF-*κ*B, JNK, and ERK1/2 signaling pathways [40]. Antiairway remodeling and hypersensitivity effect in mice model [41, 42]. Immunomodulation of Th1/Th2 balance in mice CD4 + T cell [43]. Anti-inflammation in lipopolysaccharide-stimulated RAW264.7 cells [44].
Xin-Yi-San	Immunomodulatory effect by suppressing IgE levels and increasing IL-10, sICAM-1, and IL-8 in the clinical trials [45]
Cang-Er-San	Anti-inflammation by inhibiting IL-4 and TNF-*α* in human mast cell lines [46]
Ge-Gen-Tang	Antiviral activity against the human respiratory syncytial virus [47] and influenza virus in human respiratory tract cell lines [48]

Single herb (SH)	
*Platycodon grandiflorum* (Jacq.) A. DC.	Anti-inflammation by reducing IL-13-induced cytokines via inhibition of NF-*κ*B and MAPK signaling pathways in nasal epithelial cells [49]. Anti-inflammation/ antiairway remodeling effect in mice and A549 cells [50]. Antiallergic effect by reducing IL-6, PGD (2), LTC (4), *β*-hexosaminidase, and COX-2 protein in bone marrow-derived mast cells [51].
*Glycyrrhiza uralensis* Fisch.	Anti-inflammation in a murine model of the allergic airway [52]. Antiviral activity against the human respiratory syncytial virus by preventing viral attachment, internalization, and by stimulating IFN secretion in human respiratory tract cell lines [53]. Antibacterial effect [54] and synergistic antimicrobial activity against strains of MRSA [55].
*Angelica dahurica* Benth. et Hood. F.	Anti-inflammation by inhibiting nitric oxide production in LPS-activated RAW264.7 cell lines [56]. Antiasthmatic effect by upregulating heme-oxygenase-1 in the mice model [57]. Antibacterial effect on stains of MRSA.
*Scutellaria baicalensis* Georgi	Anti-inflammation of baicalin by blocking JAK2-STAT5 and NF-*κ*B signaling pathways in LPS-stimulated human mast cells [58]
*Fritillaria thunbergii* Miq.	Anti-inflammation of peiminine by inhibiting IL-6, IL-8, TNF-*α*, and IL-1*β* via blocking NF-*κ*B and MAPK signaling pathways in HMC-1 cells [59]

## Data Availability

The data used to support the findings of this study are included within the article.

## Supplementary Materials

https://reactome.org/ Supplementary Materials Supplementary 1: The composition of all herbal formula (HFs) in the Chinese herbal medicine (CHM) network. The ratio of each composition in HF is presented as a percentage of the total weight of the prescription (S1.CHMcomposition.csv). Supplementary 2: The list of Chinese herbal medicine (CHM) and ingredients categorized by different CHM clusters in the network. Abbreviations: MW, molecular weight; HBA, hydrogen bond acceptor counts; HBD, hydrogen bond receptor counts; RB, rotatable bond counts; TPSA, topological polar surface area (S2.CHMingredients.csv). Supplementary 3: The list of western medicine (WM) evaluated in this study. Each WM was categorized into different groups by its pharmacological effect (S3.WM.csv). Supplementary 4: The list of total binding proteins of each cluster. The proteins were obtained from TCMSP, TCMID, and TCM Database@Taiwan, and the binding between CHM ingredients or WMs and proteins were queried from the STITCH and BindingDB database (S4.BindingProtein.csv). Supplementary 5: The list of all proposed pathways of each cluster. These potential pathways are introduced by the enrichment analysis in the Reactome () (S5.Pathway.csv).
